# Treatment Adherence and Factors Influencing It in End-Stage Renal Disease Patients on Maintenance Hemodialysis: A Study From a Tertiary Care Hospital in North India

**DOI:** 10.7759/cureus.73335

**Published:** 2024-11-09

**Authors:** Ankit LNU, Gaurav Rathee, Rohit Sharma, Shaveta Dahiya, FNU Vaibhav, Pardeep Kumar

**Affiliations:** 1 Nephrology, Pandit Bhagwat Dayal Sharma Post Graduate Institute of Medical Sciences, Rohtak, IND; 2 Internal Medicine, Pandit Bhagwat Dayal Sharma Post Graduate Institute of Medical Sciences, Rohtak, IND

**Keywords:** adherence to treatment, chronic kidney disease (ckd), end stage renal disease (esrd), maintenance hemodialysis, nephrology

## Abstract

Background

Chronic kidney disease (CKD) has become a significant global health concern, making its effective management crucial. Patients with end-stage renal disease (ESRD) often require multiple medications, restriction of fluid and diet intake, and maintenance hemodialysis (HD) to manage their condition, which together makes it difficult to maintain adherence to treatment. This study aimed to evaluate the prevalence of treatment adherence among ESRD patients and to identify influencing factors, addressing the lack of such data from Northern India.

Methods

This cross-sectional study was conducted at a tertiary care hospital in Northern India. ESRD patients on maintenance HD for at least three months were recruited from the outpatient and dialysis units of the hospital. Patients were interviewed using a validated and reliable tool, the End-Stage Renal Disease Adherence Questionnaire (ESRD-AQ). The primary objective was to assess the prevalence of treatment adherence among ESRD patients on HD who presented to the hospital, and the secondary objective was to evaluate various factors influencing treatment adherence among these patients.

Results

A total of 199 patients were enrolled, with a mean age ± standard deviation of 44.34 ± 13.7 years. Among the patients, 51.76% (n = 103) demonstrated good adherence (adherence score >1000), 41.71% (n = 83) showed moderate adherence (adherence score 700-999), and 6.53% (n = 13) exhibited poor adherence (adherence score <700). A significant association was found between adherence scores and patients' perception of treatment regimens (p < 0.0001 for each of the four treatment adherence domains, namely, HD, medication, fluid restriction, and diet restriction) and between adherence scores and the frequency of counseling by medical professionals (p = 0.106, 0.037, 0.014, and 0.002 for HD, medication, fluid restriction, and diet restriction, respectively). Individuals with graduate-level education or higher exhibited significantly better adherence scores than those with education levels below graduation (p = 0.044). However, age, sex, marital status, area of residence, employment status, mode of transportation, and the presence of family members accompanying patients to HD sessions were not significantly associated with treatment adherence.

Conclusion

Approximately half of the study population exhibited moderate-to-poor adherence, emphasizing the need for substantial improvement in adherence levels. These findings suggest that more frequent and effective counseling is necessary, as low adherence scores were significantly associated with infrequent counseling and poor patients' perceptions of the importance of treatment regimens.

## Introduction

“Chronic kidney disease (CKD) is defined as abnormalities of kidney structure or function, present for a minimum of 3 months, with implications for health” [1]. CKD is classified into five categories, with category 5, known as end-stage renal disease (ESRD), representing the most advanced phase of the condition [1]. For ESRD patients, renal replacement therapy (RRT) is essential for survival and includes options for long-term dialysis or kidney transplantation [2,3]. Among these options, kidney transplantation is regarded as the optimal treatment option for individuals with ESRD [4,5]. Nonetheless, because of the scarcity of organ donors, maintenance hemodialysis (HD) continues to be a more feasible treatment for ESRD patients.

ESRD patients on maintenance HD constitute a distinct demographic population concerning treatment adherence, primarily due to the complexity of their treatment regimens, which significantly influences various aspects of their daily lives. Optimal management of these patients necessitates the integration of multiple treatment domains, including timely HD sessions, medication adherence, and strict control of fluid and dietary intake. Furthermore, achieving favorable health outcomes requires lifelong behavioral modifications and social adjustments to accommodate the demands of complex treatment regimens [6].

Among patients undergoing HD, the overall mortality rate is considerably higher, estimated to be 6.3 to 8.2 times that of the general population. Factors that significantly impact survival rates include the adequacy of HD sessions, level of medication adherence, and restrictions regarding fluid and dietary intake [7]. Patients receiving long-term HD share responsibility with their treating medical professionals for the effectiveness of the treatment by following all the related advice.

Therefore, it is essential to raise awareness among patients about the importance of properly adhering to treatment regimens for better health outcomes. Non-adherence often arises from patients' reluctance to follow medical recommendations or a lack of understanding of their treatment [8]. Several studies have documented varying levels of treatment adherence across populations. These findings suggest that adherence is influenced by factors such as family support, counseling, and socioeconomic conditions, which can vary significantly across cultural and demographic contexts. Despite known challenges in treatment adherence in ESRD patients on HD, limited research exists on adherence levels in Northern India. This study aimed to estimate the prevalence of treatment adherence among ESRD patients on HD at a tertiary care hospital in Northern India and to explore factors influencing treatment adherence within this patient population.

A standardized approach for evaluating adherence levels is lacking. However, several studies have used the Dialysis Diet and Fluid Non-Adherence Questionnaire (DDFQ) and ESRD-AQ to measure adherence among ESRD patients. In a meta-analysis of 29 studies, both the DDFQ and ESRD-AQ demonstrated a strong concurrent validity when their outcomes were compared with biochemical parameters that assess adherence to dietary and fluid restrictions [9]. While the DDFQ provides information on diet and fluid adherence, the ESRD-AQ measures adherence to all four domains of treatment regimen including HD, medication, diet, and fluid restrictions, and provides insights on patient perception and other factors affecting adherence [10,11]. Therefore, this study intended to use the ESRD-AQ.

## Materials and methods

Study design

This cross-sectional, observational study was conducted from January to June 2024 at Pandit Bhagwat Dayal Sharma Post Graduate Institute of Medical Sciences, Rohtak, a tertiary care hospital in Haryana, Northern India. ESRD patients were enrolled from the outpatient and dialysis units of the Department of Nephrology of the hospital. Inclusion criteria were patients aged 18 years or older, those on maintenance HD for at least three months, and individuals able to understand and speak English and/or Hindi. Patients who were critically ill or unable to communicate were excluded from the study. The sample size was calculated using Cochran’s formula, based on adherence rates reported in a recent similar study conducted by Anuja et al. in Southern India [12]. With a 6.5% margin of error and a 5% level of significance, the minimum required sample size was determined to be 191 patients. The study was completed by 199 patients who met the inclusion criteria and agreed to participate.

Ethical considerations

The study protocol was approved by the Research Project Advisory Committee of the institute on 09/08/2023 (UHSR/RPAC/2023/287), and permission was obtained from the Biomedical Research Ethics Committee of the institute on 18/10/2023 (BREC/23/545). This study adhered to the ethical standards of the Declaration of Helsinki. Permission to use the ESRD-AQ tool in this study was obtained from the author of the questionnaire. After explaining the purpose of the study, a patient information sheet was provided to all participants, and written informed consent was obtained from each participant.

Data Collection and tools

Patients were interviewed face-to-face by the investigators, and responses were documented regarding socio-demographic details, HD session attendance and timings, taking prescribed medicines on schedule, and following fluid and dietary restrictions. Patients were asked to recall about the most recent week and month and to answer the question. The ESRD-AQ was used to estimate the prevalence of treatment adherence in the study population. This questionnaire consists of 46 questions and is a validated and reliable tool. It was organized into five categories: general information (5 questions), adherence to hemodialysis treatment (14 questions), medication adherence (9 questions), adherence to fluid restrictions (10 questions), and dietary adherence (8 questions). The overall adherence behavior was calculated by summing the scores from question numbers 14, 17, 18, 26, 31, and 46, yielding a total score range of 0 to 1200, with higher scores reflecting better adherence levels [10]. The HD adherence score was calculated by summing the scores from question number 14 (HD session attendance),17 (frequency of HD shortening), and 18 (average duration of HD session shortening), constituting a score of 0-600. Responses to question numbers 26, 31, and 46 correspond to the medication adherence score (0-200), fluid restriction adherence score (0-200), and dietary restriction adherence score (0-200), respectively.

Translation of questionnaire

Initially, the English questionnaire was translated into Hindi. Subsequently, this Hindi version was back-translated into English, allowing for a comparison with the original English version to check for consistency. In cases where discrepancies were identified, the translation was revised until both versions matched, thus ensuring lexical equivalence. Once the translation process was completed, the Hindi version was validated by specialists in community medicine and internal medicine.

Statistical analysis

Data were entered into Microsoft Excel (Microsoft Corporation, Redmond, USA) spreadsheets and analyzed using the Statistical Package for Social Sciences (SPSS) software (version 25.0; IBM Corp., Armonk, USA). Categorical variables are presented as numbers and percentages, while quantitative data are presented as mean ± standard deviation and median with interquartile range (25th and 75th percentiles). The Shapiro-Wilk test was used to check the normality of the data. Associations between quantitative variables were analyzed using the Independent t-test (for two groups) and ANOVA (for more than two groups). Statistical significance was set at p<0.05.

## Results

The study included 199 participants with a mean age of 44.34 ± 13.7 years (range: 18-80 years). Of the total participants, 61.8% (n=123) were male, 85.4% (n=170) were married, and 61.3% (n=122) resided in rural areas. In terms of education, 16.1% (n=32) were graduates or above and the remaining(83.9%, n=167) were undergraduates. Personal transportation was used by 41.7% (n=83) of the participants, while the remainder used public transportation. A total of 17.6% (n=35) of patients attended dialysis sessions unaccompanied by family members or attendants. 24 patients were working while being on ESRD treatment, including HD. Hypertension (73.9%, n=147) and diabetes mellitus (19.1%, n=38) were the two most common comorbidities observed among the study participants. The mean duration for which patients had been undergoing dialysis was 22.04 ± 26.7 months. The descriptive characteristics of the study population are shown in Table 1.

**Table 1:** Descriptive characteristics of the study population

Demographic characteristics	Number of patients	Percentage
Age		
18 to 20 years	4	2.01%
21 to 30 years	31	15.58%
31 to 40 years	45	22.61%
41 to 50 years	56	28.14%
51 to 60 years	34	17.09%
61 to 70 years	21	10.55%
71 to 80 years	8	4.02%
Mean ± SD	44.34 ± 13.7	
Median(25th-75th percentile)	44(35-52)	
Range	18-80	
Gender		
Female	76	38.19%
Male	123	61.81%
Marital status		
Married	170	85.43%
Single/Widowed/Divorced	29	14.57%
Educational status		
Illiterate	37	18.59%
Primary	38	19.10%
Secondary	62	31.16%
Senior secondary	30	15.08%
Graduate	29	14.57%
Post graduate	3	1.51%
Area of residence		
Rural	122	61.31%
Urban	77	38.69%
Employment		
Employed	24	12.06%
Unemployed	175	87.94%
Annual Income		
Below 5 lacs.	198	99.50%
> 5 lacs	1	0.50%
Mode of transport		
Personal transport	83	41.71%
Public transport	116	58.29%
Attendance at Hemodialysis Sessions		
Alone	35	17.59%
Accompanied by someone	164	82.41%
Duration for which patients had been undergoing dialysis(in months)		
Mean ± SD	22.04 ± 26.7	
Median(25th-75th percentile)	11(4-30)	
Range	3-158	
Presence of comorbidity		
Hypertension	147	73.87%
Diabetes mellitus	38	19.10%
Hypothyroidism	16	8.04%
Chronic hepatitis carrier(HBV, HCV)	9	4.52%
Cystic kidney disease	5	2.51%
None	16	8.04%

**Figure 1:**
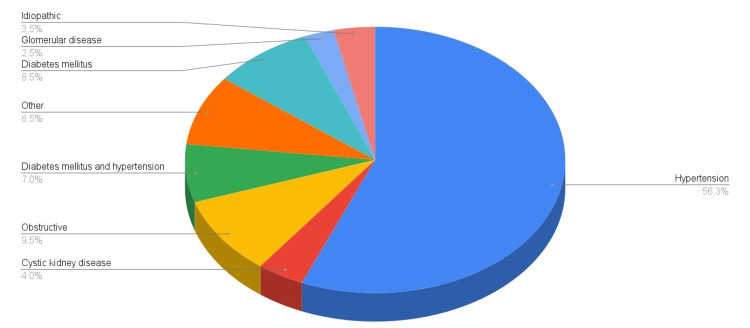
Relative contributions of various conditions as a cause of chronic kidney disease.

The most common causes of CKD in the study population were hypertension and diabetes mellitus. The relative contributions of various conditions that caused CKD in the study population are illustrated in the pie chart in Figure 1. Two patients had previously undergone kidney transplantation and were later started on HD. Four patients had previously received peritoneal dialysis and later switched to HD.

Primary outcome

The mean overall adherence score was 954.65 ± 182.6 (maximum score 1200). For specific adherence behaviors, the mean score for HD adherence was 466.71 ± 121.96 (maximum score 600), adherence to medications was 178.14 ± 42.49 (maximum score 200), fluid regimen adherence was 156.03 ± 45.38 (maximum score 200), and diet regimen adherence was 153.77 ± 57.28 (maximum score 200) (Table 2). This study found that 51.76% (n=103) of the participants demonstrated good adherence, 41.71% (n=83) exhibited moderate adherence, and 6.53% (n=13) showed poor adherence (Table 3).

**Table 2:** Overall adherence and specific domains adherence scores of the study population

Adherence score	Mean ± SD	Median(25th-75th percentile)	Range
Hemodialysis adherence score	466.71 ± 121.96	475(412.5-600)	0-600
Medication adherence score	178.14 ± 42.49	200(150-200)	0-200
Fluid regimen adherence score	156.03 ± 45.38	150(150-200)	0-200
Diet regimen adherence score	153.77 ± 57.28	150(150-200)	0-200
Overall adherence score	954.65 ± 182.66	1000(837.5-1075)	0-1200

**Table 3:** Prevalence of adherence among study population

Adherence score	Frequency (n)	Percentage
Poor adherence {<700}	13	6.53%
Moderate adherence {700 to 999}	83	41.71%
Good adherence {>=1000}	103	51.76%
Total	199	100.00%

Secondary outcomes

Effects of Descriptive Characteristics on Treatment Adherence

On examining the factors influencing adherence behaviours among the study population, age, sex, marital status, area of residence, employment status, mode of transport, and family members accompanying the patients for HD sessions were not found to significantly affect treatment adherence. Individuals with at least a graduate education demonstrated significantly better adherence scores than those with lower levels of education (p = 0.044). A slightly negative correlation (Spearman coefficient -0.144, p = 0.042) was observed between the duration of HD treatment (in months) and adherence scores, suggesting that a longer duration of dialysis may be associated with lower adherence. The association between adherence scores and various parameters is shown in Tables 4, 5.

**Table 4:** Association of overall adherence score with various descriptive characteristics † Independent t test

Variable	Mean score + SD		p-value		t-value
Gender					
Male	956.1 ± 194.53		0.887†		0.142
Female	952. ± 162.85				
Marital status					
Married	955 ± 175.28		0.948†		0.066
Unmarried	952.59 ± 224.74				
Education					
Upto senior secondary	943.26 ± 185.78		0.044†		2.024
Graduate/Postgraduate	1014.06 ± 154.75				
Area of residence					
Rural	952.05 ± 180.15		0.801†		0.252
Urban	958.77 ± 187.69				
Employment status					
Employed	926.04 ± 220.98		0.495†		0.691
Unemployed	958.57 ± 177.16				
Mode of transport					
Personal transport	951.51 ± 185.62		0.838†		0.205
Public transport	956.9 ± 181.29				
Attendance at haemodialysis session					
Alone	982.14 ± 161.17		0.328†		0.981
Accompanied by someone	948.78 ± 186.85				

**Table 5:** Spearman’s rank correlation for continuous variables with overall adherence scores

Variable	p-value			Correlation coefficient
Patient age				
Units in years	0.084			-0.123
Duration for which patients had been undergoing dialysis				
Units in months	0.042			-0.144

Frequency of Counseling by Medical Professionals

**Figure 2:**
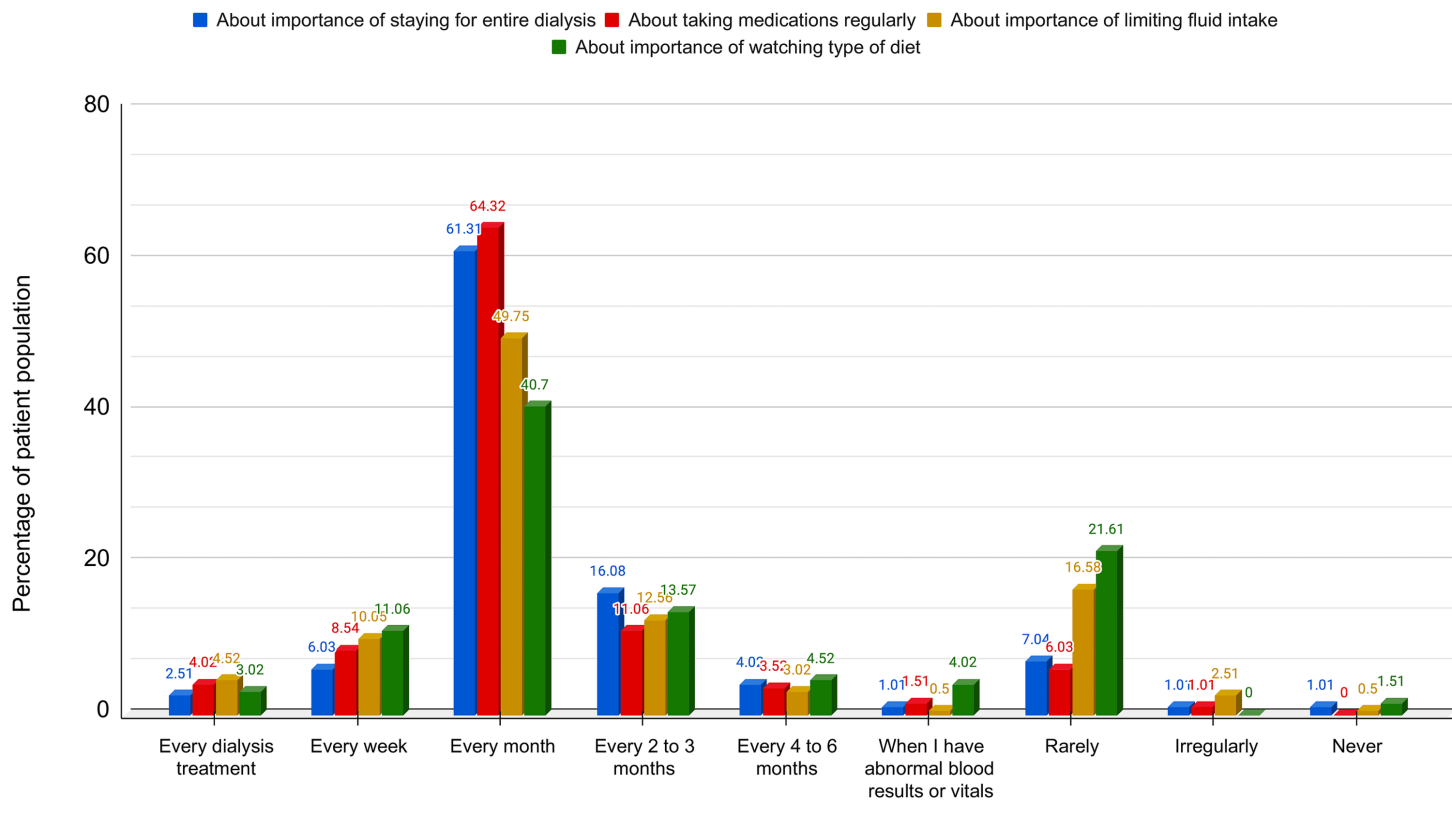
Frequency with which medical professionals discussed the importance of treatment regimens

The frequency with which medical professionals discussed the importance of various domains of treatment regimens with the patients is illustrated in Figure 2. This study found that adherence scores improved with increased frequency of counseling of patients by medical professionals regarding the importance of all domains of treatment (p=0.106, 0.037, 0.014, and 0.002 for HD, medication, fluid regimen, and diet regimen adherence scores, respectively). The association of adherence scores across all domains of treatment with the frequency of counseling is depicted in Table 6.

**Table 6:** Association between adherence scores across various domains and the frequency of medical professionals discussing the importance of treatment regimens with patients † Independent t test

Adherence score	Within 3 months	Later than 3 months	Total	p-value	t-value
Dialysis Adherence Score Mean ± SD (n)	472.37 ± 115.72 (171)	432.14 ± 152.73 (28)	466.71 ± 121.96	0.106†	1.625
Medication Adherence Score Mean ± SD (n)	181.43 ± 38.81 (175)	154.17 ± 58.82 (24)	178.14 ± 42.49	0.037†	2.206
Fluid regimen Adherence score Mean ± SD(n)	161.11 ± 41.05 (153)	139.13 ± 54.68 (46)	156.03 ± 45.38	0.014†	2.521
Diet regimen adherence score Mean ± SD (n)	163.24 ± 48.78 (136)	133.33 ± 68.39 (63)	153.77 ± 57.28	0.002†	3.122

Patient Perception of the Importance of Treatment Regimens

**Figure 3:**
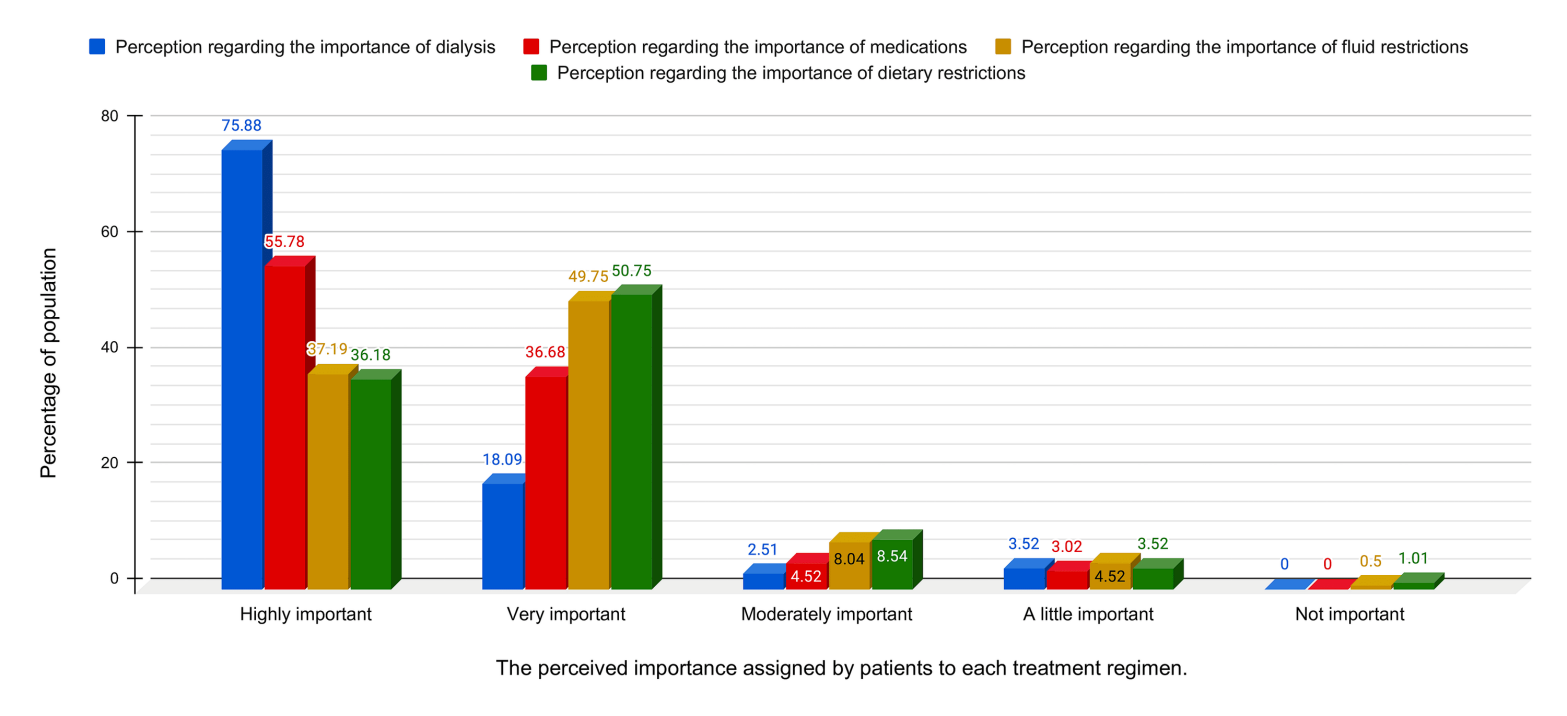
Patient perception regarding various treatment regimens

Figure 3 illustrates the frequency of patients' perceptions of the importance of various treatment regimens. This study found that the adherence score across all domains improved with more importance assigned by patients to each treatment regimen (p < 0.0001 across all domains of treatment), as shown in Table 7.

**Table 7:** Association between adherence scores in various domains with importance assigned to the treatment regimen by the participants. * ANOVA was used for more than two variables.

Adherence score	Highly important	Very important	Moderately important	A little important	Not important	Total	p-value	F-value
Dialysis adherence score Mean ± SD (n)	480.46 ± 106.18 (n=151)	457.64 ± 127.17 (n=36)	410 ± 181.66 (n=5)	257.14 ± 182.98 (n=7)	(n=0)	466.71 ± 121.96	< .0001*	8.91
Medication adherence score Mean ± SD (n)	188.29 ± 30.12 (n=111)	175.34 ± 39.2 (n=73)	122.22 ± 71.2 (n=9)	108.33 ± 86.12 (n=6)	(n=0)	178.14 ± 42.49	< .0001*	15.657
Fluid regimen adherence score Mean ± SD (n)	175 ± 39.91 (n=74)	157.58 ± 35.26 (n=99)	109.38 ± 37.5 (n=16)	83.33 ± 43.3 (n=9)	0 ± 0 (n=1)	156.03 ± 45.38	< .0001*	23.635
Diet regimen adherence score Mean ± SD (n)	172.22 ± 44.32 (n=72)	155.45 ± 54.27 (n=101)	108.82 ± 61.83 (n=17)	92.86 ± 60.75 (n=7)	0 ± 0 (n=2)	153.77 ± 57.28	< .0001*	12.414

## Discussion

Based on the overall adherence scores of ESRD patients on HD estimated using the ESRD-AQ tool in this study, 51.76% of patients demonstrated good adherence, 41.71% showed moderate adherence, and 6.53% exhibited poor adherence. In similar studies from Southern India, Anuja et al. reported good adherence in 83.3%, moderate adherence in 14.6%, and poor adherence in 2% of patients from Puducherry, while Sheeraf et al. found good, moderate, and poor adherence in 80.7%, 17%, and 2.3%, respectively, in Kerala [12,13]. The difference in adherence prevalence noted in this study compared to those in studies from Southern India suggests a significant scope for improvement in adherence behaviors. In Palestine, Naalweh et al. observed good, moderate, and poor overall adherence in 55.5%, 40.5%, and 4.1% which closely mirrors the findings of this study [14]. A more divergent pattern was observed in Pakistan, where only 10.1% of patients demonstrated good adherence, 31.9% had moderate adherence, and 58% had poor adherence [15]. In a recent meta-analysis of 23 studies published worldwide between 2000 and 2020, comprising a total of 11,209 patients on HD, non-adherence to dietary restrictions was reported, with a prevalence rate of 60.2% (95% CI: 47.3-72.5) [16]. The same study reported a prevalence of non-adherence to fluid restrictions at 60.6% (95% CI: 50-70.7) [16].

In the study, 8.04% (n=16) participants missed one HD session and 8.54%(n=17) participants missed more than one HD session during the month prior to the interview. Additionally, 49 participants shortened their HD sessions once and 71 participants shortened their HD sessions more than once by more than 10 minutes during the past month from the date of the interview. Only 75.88% (n=151) of the participants considered staying for the entire dialysis session as "highly important", according to the perception scale, while 7.04% (n=14) mentioned that they had "rarely" discussed the importance of completing an HD session with a medical professional. As non-adherence to HD treatment is a stronger predictor of mortality and overall outcome compared to non-adherence in other domains, more weight has been given to HD adherence as compared to other domains for calculating adherence scores in the ESRD-AQ tool [10,17]. Thus, missing or shortened HD sessions significantly affected the overall adherence score.

Furthermore, we noted that 8.55% of patients (n=17) reported missing medications half of the time or more frequently during the previous week. 55.78% (n=111) rated the importance of taking medication regularly as "highly important" on the perception scale. Furthermore, 6.03% (n=12) indicated that they "rarely" discussed medication adherence with medical professionals. In terms of fluid adherence, 38 patients reported not adhering to fluid restrictions for at least half the time during the previous week, while 13 patients stated that they did not understand how to adhere to fluid restrictions. Additionally, 167 participants had not weighed themselves at home in the past week and 156 participants did not view weighing outside the dialysis unit as important for managing their condition. Furthermore, 33 patients reported "rarely" discussing fluid restrictions with a medical professional, and only 37.19% (n=74) recognized fluid restriction as "highly important" according to the perception scale. The mean dietary adherence score was 153.77 ± 57.28, which was the lowest among all adherence categories. A total of 107 patients reported ignoring dietary restrictions at least half the time during the interview week, and 13 patients indicated a lack of understanding of the appropriate diet to follow. Furthermore, 43 patients stated that they "rarely" discussed dietary restrictions with medical professionals. Notably, only 36.18% (n=72) considered adherence to dietary restrictions to be "highly important" on the perception scale.

All the above-mentioned findings of this study indicate a low level of adherence across all treatment domains among patients with ESRD patients on HD. A significant factor influencing this adherence is the perception of treatment importance among patients, as well as the frequency of counseling provided by medical professionals.

There was no significant association between adherence and age or sex in this study. The mean adherence score for males was 956.1 ± 194.53 and 952.3 ± 162.85 for females. In contrast, Naalweh et al. found that older patients and men had higher odds of adherence [14]. A study conducted in Turkey found that men were 2.074 times more likely to be non-adherent to HD treatment [18]. Good adherence in females was reported by Alhamad et al from S. Arabia [19]. While a study by Dantas et al found that an age of more than 50 years to be associated with non-adherence. Marital status did not seem to affect adherence in this study, unlike Dantas et al., who reported that unmarried status exacerbated non-adherence [20]. Additionally, rural residence did not influence adherence in this study. However, Naalweh et al. found statistically significantly better adherence among city residents [14].

No association was found between adherence and mode of transport, which is consistent with the findings of Naalweh et al [14]. In contrast, a study from Pakistan reported an association which was statistically significant between adherence and access to personal transportation [15]. Additionally, Alhamad et al. found that individuals using family cars for transportation demonstrated better adherence [19].

Someone accompanying the patient for dialysis did not significantly affect adherence in this study. However, Alhamad et al. found that adequate family and social support improved adherence, while Dantas et al. found poor adherence in those attending dialysis without a companion [19,20]. Also, the findings of this study showed no association between adherence and employment status, which is similar to the findings of Alhamad et al. [19].

In terms of comorbidities, 19.10% of the patients had diabetes mellitus and 73.87% had hypertension. Naalweh et al. reported that 39% and 55% of their study population had diabetes and hypertension, respectively (sample size: 220) [14]. An Indian study found diabetes in 58.3% and hypertension in 41.6% of the patients (sample size: 60) [21]. In a study from Pakistan, 6.7% of the patients had diabetes and 36.1% had hypertension (sample size: 119) [15]. These findings indicate a variability in the prevalence of these comorbidities across different populations.

On subgroup analysis, it was found that the mean adherence was higher among individuals with higher education levels (graduation and above: 1014.06 ± 154.75 vs. below graduation: 943.26 ± 185.78), with the difference being statistically significant (p = 0.044). It was also noted that patients who received counseling by a medical professional every 3 months were found to have better adherence scores across all domains as compared to patients whose counseling was delayed beyond 3 months, although the value was not significant for HD adherence (p = 0.002, 0.014, 0.037, and 0.106 for dietary intake, fluid intake, medication, and HD session adherence, respectively). Patients who perceived their treatment regimens as "highly important" demonstrated a corresponding increase in adherence scores across all domains ( p-value <0.0001). Similarly, Naalweh et al. have also reported that better perception yields better adherence scores [14].

Thus, the results of this study suggest that patient education, frequent counselling by medical professionals, and patients' perceptions of the importance of treatment are the three most important factors affecting treatment adherence among ESRD patients on HD. Given the substantial potential for enhancing treatment adherence in this study population, frequent counseling by medical professionals, emphasizing the importance of all treatment regimens to patients, should improve perception and yield better clinical outcomes.

Study limitations

This study has certain limitations. First, the results may have been influenced by recall bias, although shorter recall periods were used to minimize recall bias. Second, the sample size was relatively small which restricts the generalizability of the findings to the broader context of North India. Third, adherence data were recorded for the last week or up to a month, which may not accurately reflect the long-term adherence patterns. Finally, 99.5% of the participants reported an income below INR (Indian Rupee) 5 lakh (500,000) per annum, with only one patient reporting an income above INR 5 lakh, therefore, we were unable to analyse and comment on any association between economic status and treatment adherence.

## Conclusions

This study found that overall adherence to treatment in ESRD patients on HD was good, moderate, and poor in 51.76%, 41.71%, and 6.53% of participants, respectively, indicating nearly half of the study population had inadequate treatment adherence. More frequent counseling by medical professionals and patients’ perceptions of the importance of treatment were found to be significantly associated with improved treatment adherence among ESRD patients on HD. These findings underscore the need for frequent targeted counseling to enhance patients’ understanding and promote better adherence to treatment regimens.
